# Phytochemical analysis and Enzyme Inhibition Assay of *Aerva javanica* for Ulcer

**DOI:** 10.1186/1752-153X-6-76

**Published:** 2012-07-31

**Authors:** Abdul Wajid Khan, Saleem Jan, Shaista Parveen, Rahmat Ali Khan, Asma Saeed, Abdul Jabbar Tanveer, Anwar Ali Shad

**Affiliations:** 1 Department of Chemistry, University of Science and Technology, Bannu, 28100, Pakistan; 2 Department of Biotechnology, University of Science and Technology, Bannu, 28100, Pakistan; 3 Department of Biological Sciences, Gomal University, D.I. Khan, Pakistan; 4 College of Veterinary Sciences, Gomal University, D.I. Khan, Pakistan; 5 Department of Agriculture Chemistry, KPK Agriculture University Peshawar, Peshawar, Pakistan

**Keywords:** *Aerva javanica*, Urease activity, Ethyl acetate fraction, Solvent-solvent extraction

## Abstract

**Background:**

*Aerva javanica* (Burm. f.) Juss. ex Schult. (Amaranthacea) is traditionally used for the treatment of wound healings, cough, diarrhoea, ulcer and hyperglycaemia. The current study was aimed to fractionate and isolate bioactive compounds and ultimately to evaluate their anti-ulcereogenic potential.

**Results:**

In order to achieve these aims, the fractionation, purifications and then biological potential determination of the isolated compounds was carried out. For purification purpose, initially extraction of the plant material was done with aqueous MeOH in the order of increasing polarity by using solvent-solvent extraction method. Phytochemical analysis revealed the presence of three compounds, 3-hydroxy-4 methoxybenzaldehyde (**1**), ursolic acid (**2**) and (*E*)-*N*-(4-hydroxy-3-methoxyphenethyl)-3-(4-hydroxy-3-ethoxyphenyl) acryl amide (**3**). Inhibition of urease activity of various fractions revealed that ethyl acetate fraction showed significant activity (*P <0.05*) as compared to other fractions. (*E*)-*N*-(4-hydroxy-3-methoxyphenethyl)-3-(4-hydroxy-3-ethoxyphenyl) acryl amide (**3**) showed marked anti ulcer activity (*P <0.05*).

**Conclusion:**

These results suggested the mild potential of *A. javanica* against ulcer.

## Background

Medicinal plants play important role in the treatment of various disorders [1,2]. The plants of genus *Aerva* (Amaranthaceae) are perennial herb under shrubs and are found in the North Temperate Zone, especially in the Mediterranean regions and Asia. About 20 species of genus Aerva are present in Pakistan and India; most of them are used in traditional medicinal system [3]. *A. javanica* (Burm.f.) Juss. ex Schult., locally known as Khar Buta, is found over a broad range of sandy sediments. Various chemical constituents including steroids, triterpenes, lipids, flavonoids, tannins, saponins, alkaloids, sulphates, carbohydrates and glycosides have been isolated from this plant [4,5]. The plant has been widely used for its therapeutic effects in relieving the swelling and pain due to kidney stones [6]. The plant decoction is used for dysentery, gonorrhea and cutaneous infections [7]. *A. javanica* showed antioxidant [8], antiviral [9], antiplasmodial [10] and antidiabetic activities [11].

Urease (urea amidohydrolase) is an enzyme that catalyzes the hydrolysis of urea to ammonia and carbamate, which is the final step of nitrogen metabolism in living organisms [12]. Carbamate rapidly and spontaneously decomposes, yielding a second molecule of ammonia. These reactions may cause significant increase in pH and are responsible for negative effects of urease activity in human health and agriculture [13,14]. Urease is responsible for urinary tract and gastrointestinal infections, possibly causing severe diseases such as peptic ulcers and stomach cancer as in the case of *Helicobacter pylori*. Ureases are also involved in the development of urolithiasis, pyelonephritis, hepatic encephalopathy, hepatic coma and urinary catheter encrustation [15,16]. Here, we investigated the antiulcer activity of different fractions as well as pure constituents of *A. javanica*.

## Results and discussion

**Figure 1:**
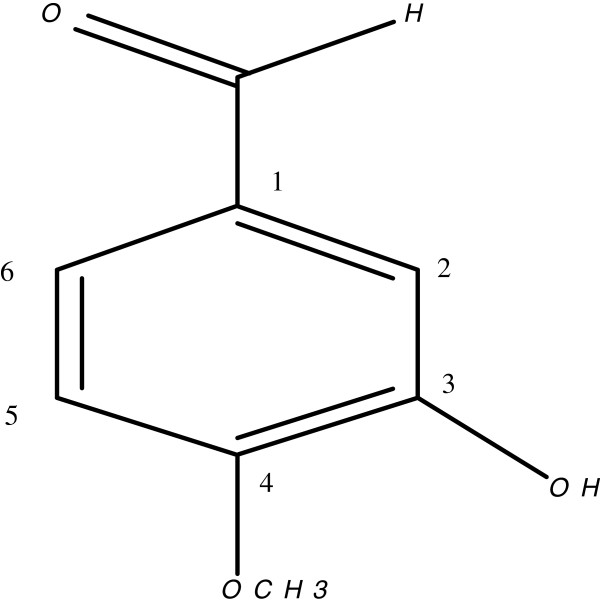
3-hydroxy-4 methoxybenzaldehyde.

**Figure 2:**
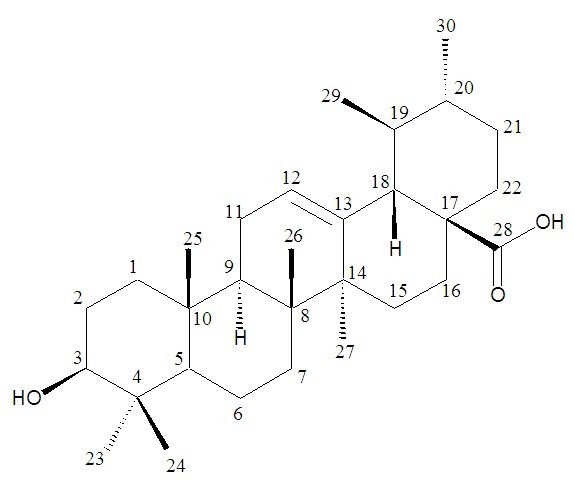
ursolic acid.

**Figure 3:**
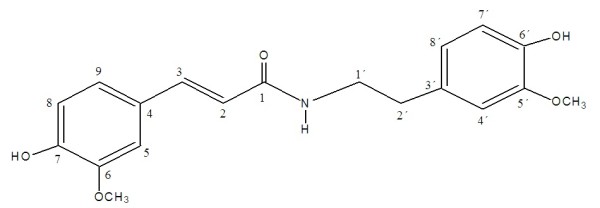
**(*E*)-*N*-(4-hydroxy-3-methoxyphenethyl)-3-(4-hydroxy-3-ethoxyphenyl) acryl amide.**

Compound **1** was isolated as white powder from the ethyl acetate fraction by *CC* and through elution with hexane: EtOAc (5:5). The HR-EIMS of **1** gave the molecular ion peak (*m/z* 152.0012) corresponding to the molecular formula C_8_H_8_O_3_ (calcd. for C_8_H_8_O_3,_ 152.0054). The IR spectrum indicated hydroxyl (3600–2500 cm^-1^), aldehydic (2685 cm^-1^), carbonyl (1705 cm^-1^) and aromatic ring (1626 cm^-1^). An aldehyde was further deduced through EIMS (*m/z* 151, M^+^-1, 100%). The ^1^HNMR spectrum of **1** (Figure 1 & Table 1) showed aldehyde (δ_H_ 9.82), three aromatic protons (δ 7.34 dd, 7.43 d and 7.1 d), hydroxyl X(δ_H_ 8.12) and a methoxy (δ_H_ 3.93, s) functionality. ^13^ C-NMR signals at δc 153.8, 148.0, 131.6, 125.1, 114.5, 111.9 further supported the proposed structure. The signal at δ_C_ 56.4 indicated –OCH_3_ funtionality. Comparison of spectral data of **1** with literature data identified compound **1** to be 3-hydroxy-4-methoxybenzaldehyde. Ursolic acid (**2**) (Figure 2 & Table 2) was obtained as colorless white crystals from the ethyl acetate fraction. IR spectrum showed strong absorptions for hydroxyl (3510 cm^-l^), carbonyl (1697 cm^-l^) and double bond (1635 and 815 cm^-1^). The molecular ion peak was at *m/z* 456.3599 in HR-EI-MS, corresponding to the molecular formula C_30_H_48_O_3_ (calcd for, 456.3603). The base peak at *m/z* 248 was due to β-type triterpenes. The HR-EIMS also exhibited a prominent peak at *m/z* 411.3640, due to the loss of COOH group. The peak at *m/z* 203.1810 was attributed to the loss of COOH from fragment at *m/z* 248.1743 at C-17, representing *retero*-Diels Alder fragmentation, which is a characteristic of Δ12 ursane type triterpene. Five tertiary methyl singlets at δ_H_ 1.20, 1.11, 0.97, 0.86, 0.82, along with two doublets at δ_H_ 1.06 (3 H, d, *J* = 6.6 Hz) and δ_H_ 0.99 (3 H, d, *J* = 6.4 Hz) in ^1^HNMR spectrum were the indication of ursane basic skeleton. The olefinic proton was at δ_H_ 5.20 (*J* = 3.5 Hz). A doublet at 2.20 ppm with *J* value of 11.3 Hz revealed that the protons at C-18 and C-19 were *trans* to eachother. Compound **3** (Figure 3 & Table 3) was obtained as yellowish white amorphous powder from EtOAc fraction. Molecular formula of **3** was established as C_19_H_21_NO_5_ by HR-EIMS, due to an ion at *m/z* 343.1411 (calcd 343.1420). The IR absorption bands at 3350 cm^-1^and 1650 cm^-1^ showed hydroxyl and amide functionalities, respectively. Highly conjugated system was exhibited due to strong absorption at 319, 290 and 220 nm in UV spectrum.

**Table 1:** ^**1**^**H and **^**13**^**C NMR spectral data for compound 1**

**Position**	^**13**^**C-NMR (δ**_**C**_**)**	^**1**^**H-NMR (δ**_**H**_**)**	***J***_**HH**_**(Hz)**
1	131.6	-	-
2	114.5	7.43	d, *J* = 2.0
3	148.0	-	-
4	153.8	-	-
5	111.9	7.12	d, *J* = 8.7
6	125.1	7.34	dd, *J* = 8.7, 2.0
OMe	56.4	3.93	s
CHO	-	9.82	s

**Table 2:** ^**1**^**H and **^**13**^**CNMR spectral data for compound 2**

**Position**	^**13**^**C-NMR (δ**_**C**_**)**	^**1**^**H-NMR (δ**_**H**_**)**	***J***_**HH**_**(Hz)**
1	38.5	1.56	m
2	27.4	1.43	m
3	79.1	3.13	dd, *J* = 10.0, 4.5
4	38.7	-	-
5	52.4	0.66	s
6	18.3	1.47	m
7	33.2	1.27	m
8	39.6	-	-
9	47.4	1.58	s
10	37.1	-	-
11	23.9	1.92	dd, *J* = 13.7, 3.5
12	125.8	5.20	t, *J* = 3.5
13	138.7	-	-
14	42.0	-	-
15	29.4	1.01	m
16	23.5	1.53	m
17	47.9	-	-
18	55.2	2.20	d, *J* = 11.3
19	30.5	1.31	m
20	30.3	1.52	m
21	27.5	1.29	m
22	37.0	1.54	m
23	24.0	1.11	s
24	15.4	0.82	s
25	15.9	0.97	s
26	17.2	0.86	s
27	24.5	1.20	s
28	176.2	-	-
29	22.4	0.79	d, *J* = 6.8
30	24.0	1.06	d, *J* = 6.6

**Table 3:** **Prominent **^**1**^**H and **^**13**^**CNMR spectral data for compound 3**

**Position**	^**13**^**C-NMR (δC)**	^**1**^**H-NMR (δ**_**H**_**)**	***J***_**HH**_**(Hz)**
Feruloyl moiety			
1	169.2	-	-
2	118.8	5.82	d, *J* = 12.8
3	142.0	6.61	d, *J* = 12.8
4	132.1	-	-
5	111.6	7.36	d, *J* = 2.0
6	149.9	-	-
7	149.0	-	-
8	116.5	6.73	d, *J* = 8.4
9	123.2	6.93	dd, *J* = 8.4, 2.0
OMe	56.4	3.82	s
Methoxytyramine moiety			
1´	42.5	3.49	t, *J* = 7.2
2´	36.2	2.77	t, *J* = 7.2
3´	128.3	-	-
4´	113.5	6.82	d, *J* = 2.0
5´	149.3	-	-
6´	146.1	-	-
7´	116.2	6.63	d, *J* = 8.0
8´	122.3	6.67	dd, *J* = 8.0, 2.0
OMe´	56.6	3.88	s

The signals at δ_H_ 6.73 (1 H, d, *J* = 8.4 Hz), δ_H_ 6.93 (1 H, dd, *J* = s 8.4,2.0 Hz) and 7.36 (1 H, d, *J* = 2.0 Hz) were for H-8, H-9 and H-5 of ferulic acid moiety, respectively. The C-3 olefinic proton of the ferulic acid moiety exhibited a doublet at δ_H_ 6.61 (*J* = 12.8 Hz) which showed the *trans*-coupling with C-2 olefinic proton, having a doublet at δ_H_ 5.82 (*J* = 12.8 Hz). A further ABX was observed at δ_H_ 6.67 (1 H, dd, *J* = 8.0.2.0 Hz) for H-8´, δ_H_ 6.63 (1 H, d, *J* = 8.0 Hz) for H-7´ and 6.82 (1 H, d, *J* = 2.0 Hz) for H-4´ in the methoxytyramine moiety. Two coupled triplets of methylene protons at δ_H_ 2.77 and δ_H_ 3.49 (each 2 H, t, *J* = 7.2 Hz) were assigned to H-2´ and H-1´, in the methoxtyramine moiety, respectively. In ^13^C-NMR spectrum, the signal at 169.2 indicated an amide functionality. ^13^C-NMR spectrum of compound **3** showed resonances of two methyls; two methylenes, eight methines and seven quaternary carbons. The mass, UV, IR and ^1^ H-NMR data proposed compound **3** an amide with phenolic acid funtionality. The position of aromatic substituent was deduced by NOESY experiment. Detailed spectroscopic data established the structure of compound **3** as the (*E*)-*N*-(4-hydroxy-3-methoxyphenethyl)-3-(4-hydroxy-3-ethoxyphenyl) acryl amide. Similarly various bioactive compounds were isolated from *A. javanica*[17].

Various fractions of *A. javanica* were tested preliminary for inhibition of jack bean urease enzyme for their potential against ulcer by using 0.2 mg/ml of each fraction and thiourea (Table 4). The data of various fractions revealed that ethyl acetate fraction of *A. javanica* exhibited significant inhibition as compared to other fractions. Therefore further chemical investigation was focused on this fraction, which led to the isolation of compounds **1**–**3**.

**Table 4:** Effects of various fractions on Urease inhibition

**Sr.#**	**Name of the fraction**	**% Inhibition**
1	0.2 mg/ml Hexane	15.3 ± 1.2^a^
2	0.2 mg/ml Dichloromethane	33.4 ± 3.2^b^
3	0.2 mg/ml Ethyl acetate	54.6 ± 4.1^c^
4	0.2 mg/ml Water	5.1 ± 0.9^a^
5	0.2 mg/ml Thiourea	98.2 ± 5.1^d^

Each value (a,b,c and d) in the table is represented as mean ± SD (n = 3).

X3-Hydroxy-4-methoxybenzaldehyde (**1**), ursolic acid (**2**), and (*E*)-*N*-(4-hydroxy-3-methoxyphenethyl)-3-(4-hydroxy-3-ethoxyphenyl) acryl amide (**3**) were screened for inhibition of urease activity (Table 5). Table data revealed that (*E*)-*N*-(4-hydroxy-3-methoxyphenethyl)-3-(4-hydroxy-3-methoxyphenyl) acryl amide (**3**) showed maximum inhibition might be responsible for antiulcer activity.

**Table 5:** Effects of isolated compounds 1–3 on Urease inhibition

**Sr.#**	**Name of the compounds**	**% Inhibition**
1	0.2 mg/ml Compound **1**	15.3 ± 0.8^a^
2	0.2 mg/ml Compound **2**	33.4 ± 2.7^b^
3	0.2 mg/ml Compound **3**	64.6 ± 4.2^c^
4	0.2 mg/ml Thiourea	98.2 ± 4.3^d^

Each value (a,b,c and d) in the table is represented as mean ± SD (n = 3).

## Experimental

### General

Electron Impact Mass Spectra (EI-MS) were measured on Finnigan MAT-311 mass Spectrometer. High-Resolution Electron Impact Mass spectra (HREI-MS) were obtained on Jeol HX mass spectrometer. IR spectra were obtained in chloroform by using JASCO IRA-1 and JASCO A-302 spectrophotometers. KBr discs as supporting suface and CHCl_3_ was used as dissolving solvent. Ultraviolet (UV) spectra of UV active compounds were recorded in methanol by using a Shimadzu UV-240 and U-3200 Hitachi spectrophotometer. The ^1^H and ^13^CNMR, COSY 45°, HMQC and HMBC spectra were recorded on Bruker AV-400 and AV-500 spectrometer.

### Plant materials

The *A. javanica* (whole plant) was collected from Bannu Township, Bannu, Khyber Pakhtunkhwa in March 2010. The plant was identified by Professor Abdur Rehman, Department of Botany, Government Post Graduate College Bannu, Pakistan and a specimen (W-12) was submitted at Herbarium of Biotechnology and Botany University of Science and Technology Bannu KPK, Pakistan.

### Extraction and isolation

The air-dried plant (20 kg) was chopped and was initially extracted with methanol/water (4:1) at room temperature. The hydromethanolic extract (700 g) was initially partitioned into *n*-hexane (84 g). The defatted MeOH extract was dried and then suspended in H_2_O (2 L), was successively partitioned with dichloromethane (110 g), and ethyl acetate (27 g). The EtOAc soluble fraction was subjected to *CC* by using normal silica gel. The eluent used were n-hexane, EtOAc, DCM and MeOH in gradient manner to obtain compounds **1**–**3**.

### Compound 1

White Powder; m.p 116–117°C; IR (KBr, CHCl_3_) *v*_*max*_ (cm^-1^): 3600–2500, 2685; ^1^H and ^13^C NMR spectral data, see Table 1; EI-MS *m/z* (rel. int.): [M]^+^, 152.0 (100), 151 (94), 137 (5), 109 (24), 95 (3), 81 (27), 43(20); HR-EI-MS: *m/z* [M]^+^ 152.0 (calcd for C_8_H_8_O_3,_ 152.05).

### Compound 2

White crystals; m.p 283–285°C; [α]_D_^20^ + 62.5° (c = 0.2, CHCl_3_); IR (KBr, CHCl_3_) *v*_*max*_ (cm^-1^): 3510, 3050, 1697, 1635, 820; ^1^H and ^13^C NMR spectral data, see Table 2; EI-MS *m/z* (rel. int.): [M]^+^, 456 (M^+^, 10), 411 (22), 248 (34), 203 (100), 189 (16); HR-EI-MS: *m/z* [M]^+^ 456.3599 (calcd for C_30_H_48_O_3,_ 456.3603).

### Compound 3

Yellowish white amorphous powder; m.p 111-113°C, IR (KBr, CHCl_3_) *v*_*max*_ (cm^-1^): 3440, 3350, 1680, 1650; ^1^H and ^13^C NMR spectral data, see Table 3; EI-MS *m/z* (rel. int.): [M]^+^, 343 (20), 193 (30), 177 (94), 151 (25), 150 (100), 145 (30), 55 (6); HR-EI-MS: *m/z* [M]^+^ 343.1411 (calcd for C_19_H_21_NO_5_, 343.1420).

#### Urease enzyme inhibition assay

Urease (Jack bean) solution (25 μl) was mixed with the 5 mg (500 μg) extracts and the mixture was incubated at 30°C. Aliquot were taken after 15 min and then was transferred to assay mixtures having urea (100 mM) in buffer (40 μl) and again incubated for 30 min in 96 well plates. Urease activity was determined by measuring ammonia production using the indophenol method as described [18]. Briefly, 50 μ1 each of phenol reagent (1% w/v phenol and 0.005% w/v sodium nitroprusside) and 70 μ1 of alkali reagent (0.5% w/v NaOH and 0.1% active chloride NaOCI) were added to each well. The increasing absorbance was measured after 50 min at wavelength of 630 nm using microtitre plate reader (Spectramax plus 384 Molecular Device, USA). All reactions were performed in triplicates in final volume of 200 μl. All the assays were performed at pH 8.2 (0.01 M K_2_HPO_4_. 3H_2_O, 1 mM EDTA and 0.01 M LiC1_2_). Thiourea was used as standard and percentage inhibitions were calculated from formula, 100 - (OD test/OD control) x 100. In this study, the reductive ability was measured by investigating the Fe^+3^ → Fe transformation in the presence of various extracts of plants and standard antioxidant (BHA) by using the Oyaizu method [19].

### Statistical analysis

The parametric data were expressed as the mean ± SEM for the 03 replicates in each group. To determine the differences between groups one-way analysis of variance (ANOVA) was carried out by using the SPSS software (version 13.0) using the least significant difference (LSD) test at *P<0.01*. Means not sharing the same letter are significantly different (LSD) at *P < 0.01* probability level in each column.

## Conclusion

The current phytochemical study provided preliminary data for the first time that the *A. javanica* possesses significant anti-ulcer activity. This might be contributed towards the presence of some bioactive constituents contributed towards the various biological activities including the in the treatment of gastric ulcer. The compound **3** shows mild activity which might be due to the presence of amide group. Further study on the plant and specifically on the compound **3** could provide many chemically interesting and biologically active drugs, including, some with potential anti-ulcer properties.

## Competing interest

The authors declare that they have no competing interests.

## Authors’ contributions

SJ has supervised all the research work carried out. AWK carried out isolation, purification and characterization of the constituents. SP and AAS facilitate in research work. RAK made a significant contribution to acquisition of data, analysis, drafting of the manuscript. AJT and AS helped in extraction. All authors read and approved the final manuscript.
